# The Structural and Thermal properties of *Badarashma Pishti*

**DOI:** 10.1016/j.jaim.2024.100989

**Published:** 2024-11-29

**Authors:** Chandrashekhar Y. Jagtap, Vaibhav Charde, Hemant Rawat, Ganesh Dane, Ashwini Kumar Mishra, Ch. Venkata Narasimhaji, Bhagwan S. Sharma, Shruti Khanduri, Ravindra Singh, Narayanam Srikanth, Rabinarayan Acharya

**Affiliations:** a Central Ayurveda Research Institute, Jhansi, Uttar Pradesh, 284003, India; b Central Council for Research in Ayurvedic Sciences, New Delhi, 110058, India

**Keywords:** Anti-urolithic, *Badarashma pishti*, Jews stone, *Lapis judaicus*, Standardization

## Abstract

**Background:**

*Lapis judaicus*, or Jews stone (*Badarashma*), has been used in treating kidney and bladder stones since ancient times by Ayurvedic and Unani medicinal practitioners. A limited number of studies have been carried out using Ayurvedic preparations derived from gems or precious stones that were used traditionally. In Ayurveda, Rasa Shastra includes the fabrication of *Bhasmas* and *Pishti*.

**Objective:**

This study aimed to prepare and characterize *Badarashma Pishti* formulation (*Lapis judaicus* microparticles) and investigate its structural and thermal properties.

**Materials and methods:**

The microparticles were prepared by subjecting purified powder of *Lapis judaicus* to a wet levigation (*Bhavana*) process. Characterizations were done using Fourier transform infrared spectroscopy (FTIR), Differential scanning calorimetry (DSC), thermo-gravimetry analysis (TGA), energy-dispersive X-ray (EDAX) spectroscopy, X-ray diffraction (XRD), and scanning electron microscopy (SEM) analysis. Physicochemical characterization, elemental analysis, particle size distribution study, and identification of heavy metals were also performed.

**Results:**

A standard operating procedure was developed to formulate *Badarashma Pishti*. The obtained microparticles were irregularly shaped with a size of 4.290 ± 0.472 μm, confirmed in SEM images. The FTIR and XRD of prepared *Badarashma Pishti* samples revealed the presence of calcite, quartz, and aragonite minerals. The results of EDAX also confirmed the presence of Ca, Mg, O, and Si elements.

**Conclusion:**

The proposed study results reveal great insights for determining the authenticity, purity, and standardization parameters of inorganic mineral drugs, proving to be a useful delivery system for treating renal calculi.

## Highlights

• Preparation and characterization of *Badarashma Pishti* (*Lapis Judaicus* microparticles).
• XRD spectrum reveals the phase transition from orthorhombic to Rhombohedral phase at temperature 787 °C.
• The FESEM images show that the prepared particles are of irregular shape and of micrometer scale.
• EDAX reveals the elemental composition of *Lapis Judaicus* microparticles.
• The study will set a standard for preparing and characterizing other precious gem microparticles.

## 1 Introduction

Nephrolithiasis is a chronic condition that can come back at any point in a patient's life, and it is imperative to take effective prophylactic measures in order to reduce the risk of future episodes [1]. Research into alternate treatment possibilities is continuing even though there is no guarantee that the kidney stones will not return. Jews stones are small necked with a globular head adorned with beaded ribs commonly known as *Lapis judaicus* (with synonyms: *Sang-e-Jahudan* in Persian, *Badarashma* in Sanskrit, *Ber patthar* in Hindi, and *Hajarul Yahud* in Arabic) with lithotritic property. *Lapis judaicus* has been used in various countries like India, Afghanistan, Iran, and Pakistan since ancient times for the management of renal stones [[2], [3], [4]]. Anti-urolithic effects have been reported by the Unani and Ayurvedic systems of medicine. Furthermore, some Iranian scholars, Dioscorides and Galen, have reported the efficacy of *Lapis judaicus* in reducing kidney stones [5,6]. This mineral drug is available in various shapes and morphologic sizes. Numerous famous scientists have written works discussing the pharmaceutical applications of pharmaceuticals derived from minerals, the processes used to purify these compounds, and the pharmacological effects they produce [4]. *Lapis judaicus* is a fossil stone mainly consisting of silicate lime with a long history of effectively dissolving renal stones and other urinary disorders [7,8]. *Lapis judaicus* is also known as *Badarashma* or ber Patthar, and its structural orientation resembles that of the Indian jujube fruit (*Ber* in Hindi). As per ancient Ayurvedic literature, “*Rasamritam"* *Badar* means *Ber,* and *Ashma* means hard stone [9]. Rasa Shastra is a branch of Ayurveda known to be an ancient Indian alchemy that deals chiefly with the Herbo-mineral preparations such as *Bhasmas* (metal micro and nanoparticles prepared by incineration process), *Rasaushadhies* and *Pishti* [10,11]. According to the Ayurvedic literature “Ayurveda Sarasamgraha," the word *Pishti* means a finely powdered drug which being triturated without the use of any heat treatment in particular liquid media (for example, decoction or rose water) till it reaches micro-sized particles. These preparations provide coolant properties to patients suffering from heat-related illnesses [[12], [13], [14]]. Herbal and mineral formulations are assessed using organoleptic, microscopic, macroscopic, and physicochemical parameters to standardize their qualitative and quantitative aspects [15,16]. In bulk materials, surface tension was found to be affected by the presence of microparticles. By altering the size scale of the particle diameters, one can easily see notable alterations in the thermodynamic properties of the materials in issue [17]. The surface-to-volume ratio (s/v) of micrometer-scale particles is negligible. Furthermore, microparticles exhibit notable variations in quantum phenomena, such as vibration path lengths and optical characteristics [18,19]. The drying process of ceramic material is influenced by the connection between particle size and form, the absorption of liquid water within the ceramic body, and the loss of moisture occurring on the surface. Various factors can contribute to process losses during drying, including issues with the conformation process (such as problems with extrusion) and characteristics of the raw material (such as shape and mineralogy) [20]. The mechanical features of clays are significantly affected by the pore fluid's chemical composition. The acid-insoluble ash and total ash of raw drug (*Lapis judaicus*) and prepared samples were also analyzed in the current study because pore fluid acidity and alkalinity affect the behaviour of clays [21].The present work provides an overview of changes in key physiochemical features that occur when transitioning from the macro to micro scale during processing, highlighting their importance for effective outcomes. As mentioned in ancient Ayurvedic textbooks like *Rasamritam* and Ayurvedic *Sarasamgraha* literature, precious gems are usually utilized in the preparation of these microparticles, such as Ruby, Pearl, Coral, Emerald, Sapphire, Topaz, Cinnamon Stone, Cat's eye, Amber, etc. Silicate-based compounds such as *Lapis judaicus*, agate, and calcium-based minerals like Oyster's shell were also utilized in the preparation of *Pishti* (microparticles providing cooling effect) [9,12,22]. In addition to this, it is an essential component in a few other Unani medications, including *Kushta Hajrul yahood, Majoon Hajrul yahood, Dawa-e-Sang, Majoon Sang Sarmahi, Safuf Hajrul yahood,* and *Majoon Yadullah.* Since limited literature on its chemical composition, physicochemical characterization, crystal lattice, and formulation approaches are available on this mineral drug, the present investigation establishes this data by preparing microparticles of *Lapis judaicus* as per ancient Ayurvedic methods. These prepared microparticles were known as *Badarashma Pishti*. In the current study, we have also characterized and compared them with the raw powder of *Lapis judaicus* using modern analytical techniques.

## 2 Materials and methods

*Lapis judaicus* (encrinite seed), an olive-shaped raw mineral drug, was purchased from Gem Herbal, Indore, India, and was authenticated by a Rasa Shastra expert. The seeds of *Dolichos biflorus* L. (*Kulthi* in Hindi) and rose water were purchased from the local market Jhansi, UP, India, and their authenticity was duly investigated by Botanists. The *Pishti* formation process involves the preparation of *D. biflorus* L. decoction, the Purification of *Badarashma* (raw form of *Lapis judaicus*), and *Badarashma pishti* (BP). Double distilled water was used throughout the whole preparation and purification process.

### 2.1 Preparation of D. biflorus L. Decoction

200g of *Dolichos biflorus* L. seeds were soaked in 1000 mL of doubled distilled water and boiled for 4–6 h to obtain decoction. The pH was 7.4 [23].

### 2.2 Preparation and purification of *Badrashma Pishti* (*Lapis judaicus* microparticles)

**Fig. 1:**
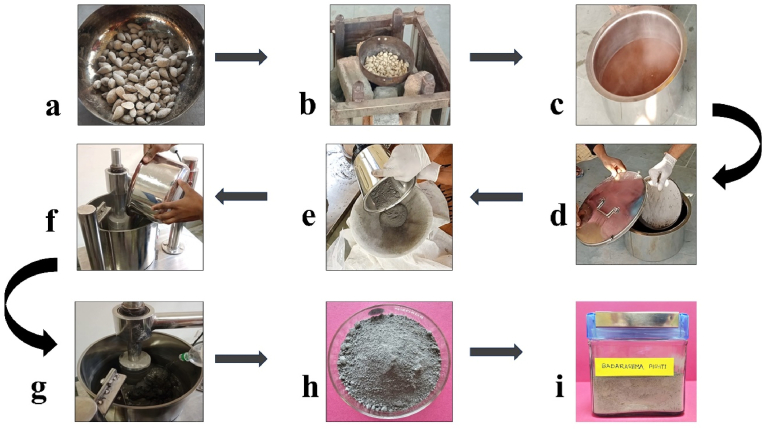
The major stages involved in the preparation of (*Badrashma Pishti*) BP showing (a) collected *Lapis judaicus* stones, (b) *Lapis judaicus* stones are heated, (c) *D. biflorus* L. decoction, (d) stones dipped in decoction to form coarse RPL, (e) RPL passed from muslin cloth (f) RPL poured in edge runner mill, (g) Rose water is added to this RPL and subjected to milling (h) drying of prepared BP (h) BP (microparticles of *Lapis judaicus*) stored in an air-tight glass container.

Olive-shaped *Lapis judaicus* stones are heated to 600 °C inside an iron vessel, causing them to break down spontaneously. The hot, broken stone pieces are dipped into a decoction made from *D. biflorus* L., which cools and forms a coarse raw powder of *Lapis judaicus* (RPL). This whole procedure was repeated ten times to remove all the impurities (process impurities, e.g., other mineral residue on ignition, foreign organic particles, etc.). This whole procedure is termed *Shodhana* (a process of removing physical and chemical impurities) in Ayurvedic literature [24]. As per the ancient Ayurvedic book “Siddha Yoga Samgraha," after the purification of the raw stones, the obtained coarse powder is subject to *Bhavana* (wet levigation) [25,26] in rose water for seven days in an edge runner mill (YS-222 York sales Pvt. Ltd. India) which results in the formation of micro-sized *Lapis judaicus* particles which were further dried (see Fig. 1). These prepared microparticles of *Lapis judaicus* were known as *Badrashma Pishti.* The samples were placed in air-tight glass bottles.

## 3 Characterization of raw drug and prepared formulation

### 3.1 Physiochemical characterization

All the physicochemical evaluations of raw powder of *Lapis judaicus* (RPL) and prepared BP samples were performed as per the methods described in Ayurvedic Pharmacopoeia of India [27]. The parameters evaluated were loss on drying, pH (10% solution), water extractive value (% w/w), alcohol extractive value (% w/w), total ash value (% w/w), acid insoluble ash value (% w/w).

### 3.2 Determination of particle size distribution

The size distribution study of the RPL and prepared BP samples were analyzed using a particle size analyzer by instrument PSA 1190, Anton Paar, Durban [28]. The D10 denotes that 10% of the volume of the sample tested comes within the range of data obtained under it. Similarly, D50 and D90 represent the sample volume tested within the range of data obtained.

### 3.3 Determination of heavy metals using ICP-OES

Heavy metals such as arsenic (As), lead (Pb), cadmium (Cd), and mercury (Hg) in the RPL and BP were performed using Inductively coupled plasma optical emission Spectrometry (ICP-OES) with the implementation of procedures according to the May 2011 amendment of *USP* 233 [29,30]. In brief, 0.5g of RPL and BP samples were mixed thoroughly in 5 mL of concentrated hydrochloric acid. 5 mL of nitric acid was added to this mixture, and the closed vessel microwave digestion method was used to obtain the clear solution. The Anton Par Microwave GO plus microwave digester was used to digest samples having 1–12 no. of vessels. The volume of each vessel was 50 mL. The filling volume must be between 3 and 25 mL, and the sample should not exceed 3g per vessel. Hydrofluoric acid resistance was used to dissolve the silicate-containing samples. A Pre-digestion of 15 min at room temperature was done in continuation of flat heating Ramp (to 1200 W, 150 °C) of 15 min and then cooled down within 30 min. Then, these digested samples were transferred in a 50 mL volumetric flask, and the volume was made up to 50 mL using Millipore water. This sample was kept aside and will be used for further analysis. A blank solution was used to calibrate the instrument to zero. Afterward, the prepared samples were inserted, followed by test samples, and the intensity was measured.

### 3.4 Elemental analysis

The samples of RPL and BP were subjected to total carbon, hydrogen, nitrogen, and sulphur (CHNS) elemental combustion system using an Elementar Vario El analyzer to determine the composition of any organic matter present. In this analysis, the RPL and BP samples 5–10 mg were mixed with vanadium pentoxide, which was sealed in a tin capsule and subjected to combustion at 1000 °C. The combustion by-products (CO_2_, SO_2_, and NO_2_) were carried away by carrier gas helium with activating catalyst tungsten trioxide and copper at 1000 °C reactor temperature. Further, these gases were separated by a 2-m packed column, and their quantification was performed by thermal conductivity detectors [31,32].

### 3.5 FTIR analysis

The samples of RPL and BP were analyzed for their vibrational spectra using the FTIR spectroscopic technique using a Bruker alpha II instrument. A potassium bromide (KBr) pellet of 13 mm in diameter and 1–2 mm in thickness, consisting of a 1 mg sample, was prepared and heated at 120 °C to minimize moisture absorption. These pellets were instantly scanned in a range of 450–4000 cm^−1^ with a resolution of 4 cm^−1^ under transmission mode [33].

### 3.6 Thermal analysis

In order to examine the impact of heat on RPL and BP, a thermogravimetric-differential thermal analysis (TG-DTA) was performed. The STA7000 apparatus, manufactured by Hitachi in Japan, was utilized for this investigation. In the TG-DTA experiment, a sample of RPL and BP weighing 5 ± 0.5 mg was subjected to heating from an initial temperature of 30 °C to a final temperature of 750 °C. The heating process was conducted at a rate of 10 °C per minute, and the environment around the sample was composed of air [34]. The temperature range for conducting heat capacity measurements with the DSC Netzsch 204 F1 with Heat flux DSC instrument is −180 to 700 °C. The heating rates employed during these studies vary from 0.001 to 200 K min-1. The purge gas utilized in the experiment was dry nitrogen, introduced at a 20 mL/min flow rate. The DSC measurement cell comprises a furnace and an integrated sensor with dedicated locations for the sample and reference pans [35].

### 3.7 XRD analysis

The presence of different minerals in BP samples was further analyzed by powder XRD (X-ray diffraction) studies using Bruker D8 Advance X-ray diffractometer with a scan speed of 0.04⁰/s. The diffraction patterns were obtained by instrument using graphite monochromatized Cu Kα radiation and recorded by step scanning operating at 40 kV and 40 mA [36]. The samples were further analyzed for their microscopic characterizations.

### 3.8 SEM-EDAX

60 mg of sample was weighed from each batch, which was immediately mixed with 6 mL of acetone and centrifuged for 10 min at 5000 rpm. The supernatant was taken and air-dried overnight at 37 °C. The samples from all three batches were placed in plastic stubs coated with carbon sputter to overcome charging disturbances. In the end, these samples were tested under the scanning electron microscope (Jeol 6390LA/OXFORD XMX N with an accelerating voltage of 0.5–30 kV) in conjugation with energy dispersive X-ray analysis (EDAX) to detect the inorganic compounds. The sample specimens were recognized by SEM operated with Vantage systems, and EDAX spectral data was gained for 100 s live time with a working distance of 20 mm [37].

## 4 Results and discussion

*Lapis judaicus* is a traditional medicine for urinary disorders in Eastern and Western countries. In addition, it is also being used to treat inflammatory bowel disease, snake bites, wounds, and stings. As the Ayurvedic and Unani literature mentions, *Lapis judaicus* dissolves urinary calculi. Previously, it was pulverized to powdered form and suspended in water for oral administration [[38], [39], [40], [41]]. Due to the lack of chemical constituents and limited physicochemical and pharmacological exploration of *Lapis judaicus*, its clinical role is limited. BP was prepared and purified using the *Shodhana* process ten times and seven repeated cycles of the *Bhavana* process. The prepared microparticles were compared with raw powder of *Lapis judaicus* (RPL) for physicochemical parameters, elemental analysis, microscopic analysis, FTIR, and XRD for crystallinity changes observed. The details of the physicochemical analysis of RPL and BP are expressed in Table 1. The obtained BP was found to be light grey in color. The weight loss on drying the BP was found to be 0.28 ± 0.02 % w/w, which was lower than RPL, showing that bacterial or fungal growth may not occur in samples. Extractive values explore various physicochemical aspects of crude drugs [42]. Higher water extractive value was responsible for minimal adulteration, proper processing during drying or storage, and the presence of water-soluble constituents in the sample [43,44]. The water-soluble extractive values of BP were found to be higher than RPL, which shows enhanced solubility of prepared formulation in water. The alcohol-soluble extractive values of BP were higher compared to RPL, which shows that BP's solubility in alcohol was improved. The ash values usually represent the quality and purity of crude drugs from plant or mineral sources [42,45,46]. The ash values are used to determine the proportion of organic impurities (phosphates and carbonates) present in the sample to be tested. The total ash values of RPL and BP were 94.24 ± 2.46 and 95.03 ± 3.02, respectively, which shows that the prepared formulation has a minimal amount of organic content as a major portion is mineral content only. Acid insoluble ash (AIA) will serve as a marker for nutritional research and indicate the presence of siliceous materials found in crude herbal and mineral drugs. The method to determine AIA comprised the parameters for a successful ashing, the parameters for an effective HCl treatment (concentration, heating temperature and duration, washing water temperature, and cycles), and the parameters for a successful quantitative recovery of AIA [47]. The AIA values of RPL and BP were lower than 5% w/w which were acceptable as per standards [48,49]. The pH of the 10% solution of RPL and BP was near about 8.3, representing the basicity of *Lapis judaicus* preparations in solution. The AIA also indicates that the solubility of prepared BP will be high in the gastric environment, similar to the result of a previous study [31].

**Table 1:** Physicochemical parameters of raw powder of *Lapis judaicus* (RPL) and *Badrashma pishti* (BP) are expressed above.

S.no.	Parameters	Raw Powder of *Lapis Judaicus* (RPL) n = 3 (±SD)	*Badarashma Pishti* (BP) n = 3 (±SD)
1.	Loss on drying (% w/w)	0.43 ± 0.02	0.28 ± 0.02
2.	pH (10% solution)	8.3 ± 1.52	8.37 ± 1.42
3.	Water soluble extractive Value (%w/w)	0.71 ± 0.04	8.37 ± 1.06
4.	Alcohol Soluble Extractive Value (% w/w)	0.76 ± 0.06	1.87 ± 0.08
5.	Total Ash Value (% w/w)	94.24 ± 2.46	95.03 ± 3.02
6.	Acid Insoluble Ash Value (% w/w)	0.93 ± 0.16	2.40 ± 0.22

**Fig. 2:**
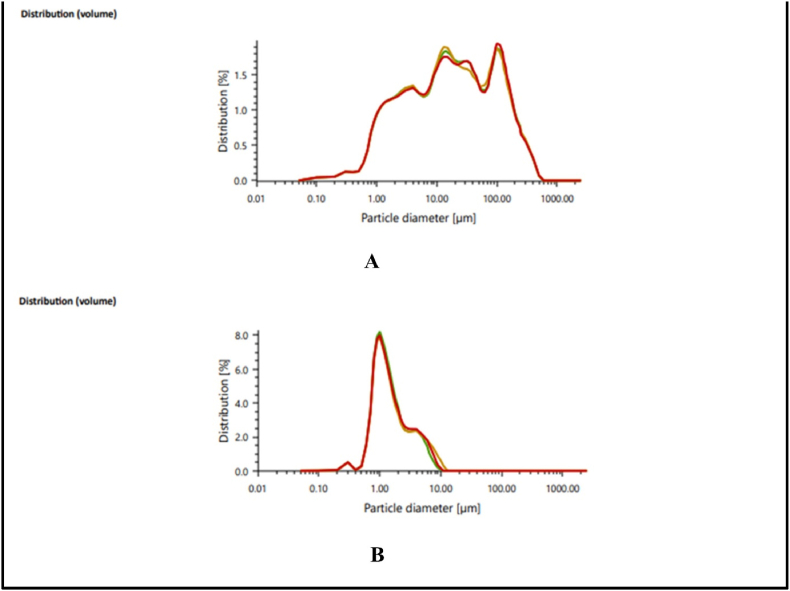
The graph shows the size distribution of (A) RPL and (B) BP.

The solubility, mouthfeel, and visual appeal of powdered materials are all factors that may be affected by particle size. The cumulative frequency curves of the RPL and BP describe the particle sizes analyzed in our present study in Table 2, by which we can conclude that the volumetric and geometric properties of both samples are variable. The D10 shows that 10% of the volume of microparticles falls between the values given under it. Similarly, D50 and D90 show that 50% and 90% of the sample volume resides within the range of values under it. The volumetric mean diameter (VMD) of RPL and BP was found to be 50.78 ± 0.540 μm and 2.16 ± 0.110 μm, respectively. The repeated cycles of *Bhavana* were used to prepare microparticles and nanoparticles [26,50,51]. It was observed that the particle size of BP was significantly decreased as compared to RPL, which was confirmed by the size distribution graph shown in Fig. 2A and B. It was also observed that after the repeated cycles of *Bhavana,* the uniformity of size was also achieved as the graph of BP (shown in Fig. 2B) shows a sharp peak compared to RPL. The stability of microparticles was also influenced by particle size constraints [52].

**Table 2:** The individual diameters of the particles determine the particle size distribution of raw powder of *Lapis judaicus* (RPL) and *Badrashma pishti* (BP).

Sample	D10 (Volume μm) n = 3 (±SD)	D50 (Volume μm) n = 3 (±SD)	D90 (Volume μm) n = 3 (±SD)	Mean (Volume μm) n = 3 (±SD)
RPL	1.298 ± 0.009	15.910 ± 0.315	135.495 ± 1.004	50.78 ± 0.540
BP	0.684 ± 0.004	1.232 ± 0.020	4.290 ± 0.472	2.16 ± 0.110

The concentrations of various heavy metals, which were As, Cd, Hg, and Pb in RPL samples, were reported as 655.59, 58.84, 0.00, and 0.71 parts per billion, respectively., The concentration of various heavy metals As, Cd, Hg, and Pb in BP samples were reported as 130.37, 217.52, 122.71and 49.82 parts per billion, respectively. The concentration of the heavy metals was found to be minimal in both RPL and BP samples. Moreover, the heavy metal concentration complied with pharmaceutical standards [[53], [54], [55]].

The CHNS elemental analysis of RPL samples was found to have 0.27 ± 0.04% nitrogen, 11.85 ± 0.52% carbon, 0.82 ± 0.06% Sulphur, and hydrogen not detected. Analysis of BP samples showed 5.41 ± 0.137% carbon, 0.153 ± 0.02% hydrogen, and no Sulphur and nitrogen detected. Patients suffering from malnutrition and acute or chronic infection often undergo catabolism, which increases the nitrogen levels in their urine [56]. Strong evidence links a high intake of animal proteins with an elevated probability of kidney stone development due to an increased sulphate load in the kidneys [57,58]. In prepared samples of BP, no nitrogen and Sulphur were detected compared to RPL, which shows that they are safe to consume and may be an effective alternative for dissolution of kidney stones.

**Fig. 3:**
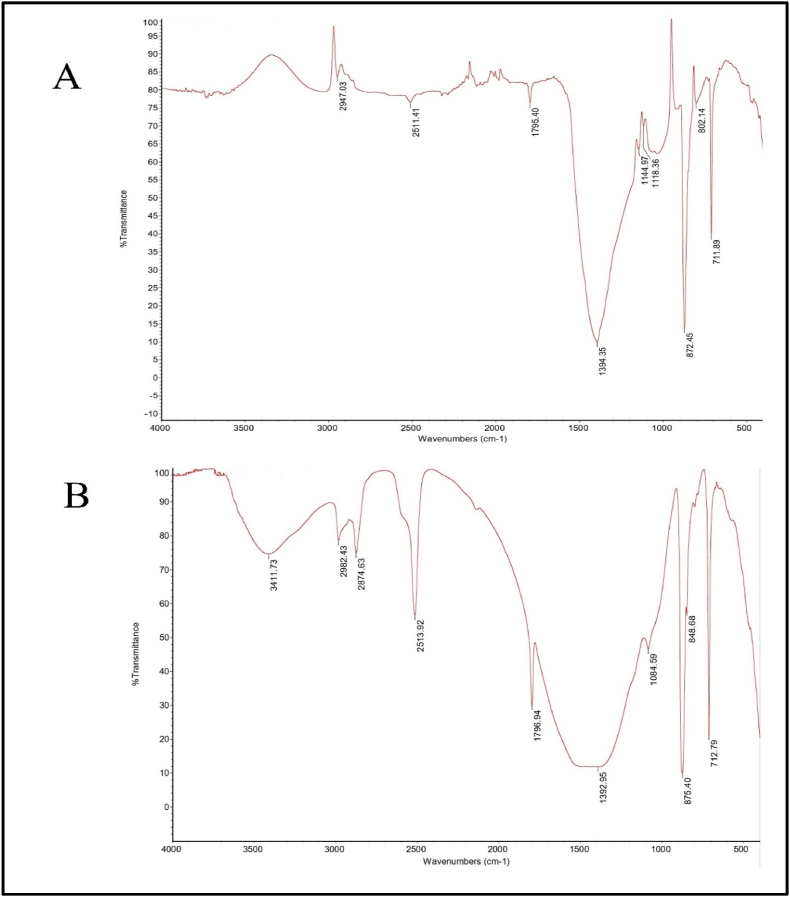
Absorption FTIR spectra of (A) RPL (B) BP.

FTIR analysis of different mixtures must be understandable for the obtained absorption bands, which reveal the sample's various physical and chemical characteristics. Such analysis is used to identify different functional groups present in a sample. Every molecule in the sample bears a unique IR spectrum except the enantiomers. The FTIR spectral analysis of the RPL and BP samples revealed different bands. The resulting bands were compared with the available literature to interpret relative functional groups [33,[59], [60], [61]]. According to the FTIR spectra of the RPL sample obtained, which were shown in Fig. 3A, give vibrational modes of the hydroxyl group at 3411.7 cm^−1,^ which was absent in the BP sample (shown in Fig. 3B). The peak observed at 2947 and 2874 cm^−1^ show the presence of organic carbon in both RPL and BP. The peaks of calcite (carbonate minerals) were observed in both samples at 1795 and 1796 cm^−1^. As per reference [62,63], the peak appeared around 1394 cm^−1^ and 1392 cm^−1^ due to mineral cerussite in both RPL and BP samples. The peaks seen at 1084 cm^−1^ in BP and 1144 cm^−1^ 1118 cm^−1^ in RPL samples are due to the Si–O asymmetrical stretch of quartz mineral present. The peaks at 711 cm^−1^, 802 cm^−1^,872 cm^−1^ in the RPL sample and 712 cm^−1^, 848 cm^−1^, and 875 cm^−1^ were observed due to the presence of calcite which was not of closed peaks of quartz (795-800 cm^−1^) [63,64]. The observed findings of FTIR analysis confirm the presence of quartz, which was in close agreement with previous literature [38].

**Fig. 4:**
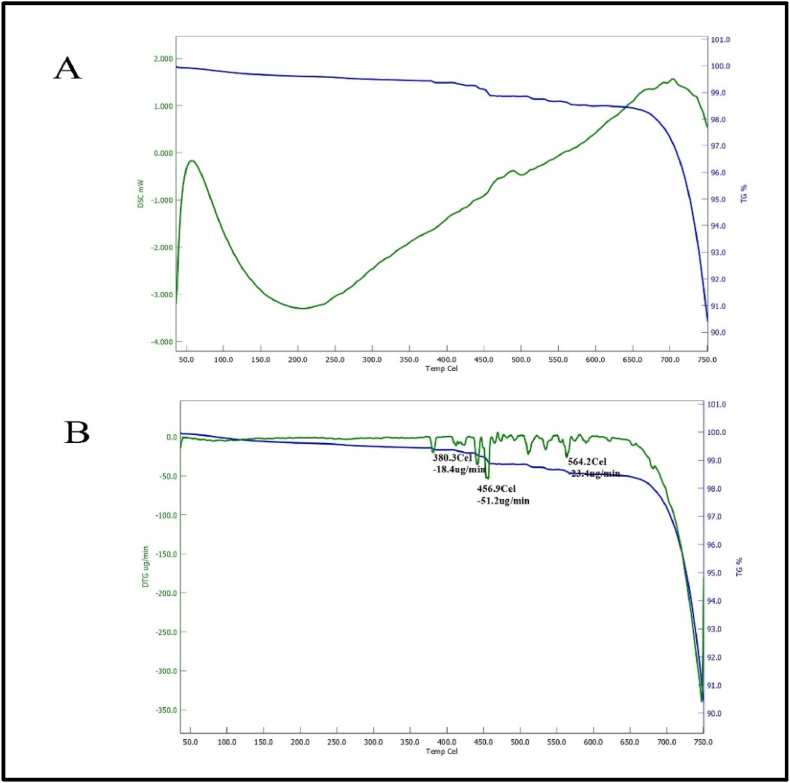
(A)TG-DSC analysis of RPL (B) DTG analysis of RPL.

The present study aimed to evaluate the decomposition properties of RPL and BP. The objective was to understand better the underlying mechanisms by which heat impacts RPL and BP. Fig. 4A depicts the change in mass of RPL that occurs during the heating process. The thermogravimetric analysis (TGA) curve revealed that the average mass loss ranged from 1% to 9% within the temperature range of 430.5 °C–750 °C. The observed mass losses might be attributed to water dehydration from the silanol (Si–OH) groups. The differential scanning calorimetry (DSC) curves exhibited a pronounced endothermic response, characterized by a peak at 196 °C, which can be attributed to the presence of silicon dioxide. The shift observed at 380.3 °C (with a weight loss of 0.6%) in the DTG curve can be ascribed to the evaporation of surface water. Additionally, the peaks observed at 456.9 and 564.2 °C (with a weight loss of 0.3%) are believed to result from silicates transforming into alternative chemical forms. As depicted in Fig. 4B, the phenomenon of buoyancy is evident throughout the heating process within the temperature range of 50–450 °C, as indicated by the continuous line. The presence of a minor endothermic peak seen at around 456 °C can likely be attributed to the dehydroxylation process of the hydroxyl water content (Ca (OH)_2_) inside the sample.

**Fig. 5:**
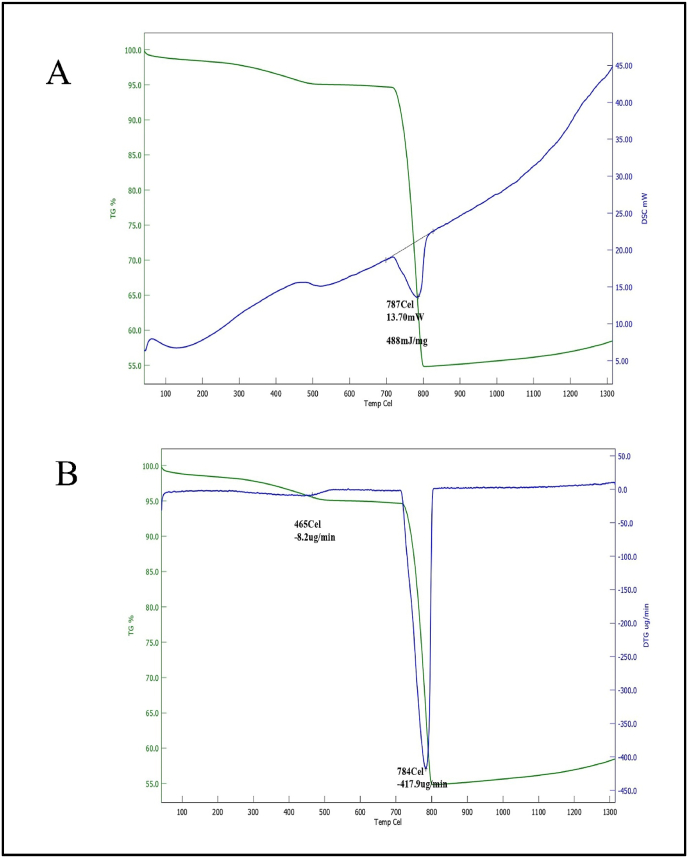
(A)TGA-DSC analysis of BP (B) TG-DTG analysis of BP.

Fig. 5A depicts the change in mass of BP that occurs during the heating process compared to the RPL sample. It was observed that the TGA curve exhibited an average mass loss of 5% within the temperature range of 170.5 °C–831 °C. The observed mass losses might be attributed to water dehydration from the silanol (Si–OH) groups. A significant decrease in the curve depicting the melting point of the produced sample was observed at temperatures of 787 °C. These temperature points corresponded to mass losses of 45% for the corresponding sample. The shift observed in the DTG curve at 465 °C (with a corresponding weight loss of 6%) can be attributed to the evaporation of surface water. Furthermore, the peak observed at 784 °C (with a weight loss of 45%) is believed to result from the transformation of silicates into alternative forms. The presence of the buoyancy effect may be noticed throughout the heating process within the temperature range of 100–830 °C, as depicted in Fig. 5B. A continuous and uninterrupted line characterizes this effect. The presence of a minor endothermic peak at about 465 °C in the observed sample was attributed to the dehydroxylation process of the hydroxyl water content (Ca (OH)_2_) inside the sample. The observed endothermic peaks occurring at a temperature of 784 °C are likely indicative of the disintegration of the magnesite structure. This disintegration process leads to the liberation of carbon dioxide from the carbonate ion associated with the calcium component of the structure. Following that, calcite and magnesium oxide develop. The differential scanning calorimetry (DSC) curves exhibited a pronounced endothermic response, characterized by peak temperatures 787 °C. This phenomenon may be attributed to the presence of silicon dioxide in the individual batches.

**Fig. 6:**
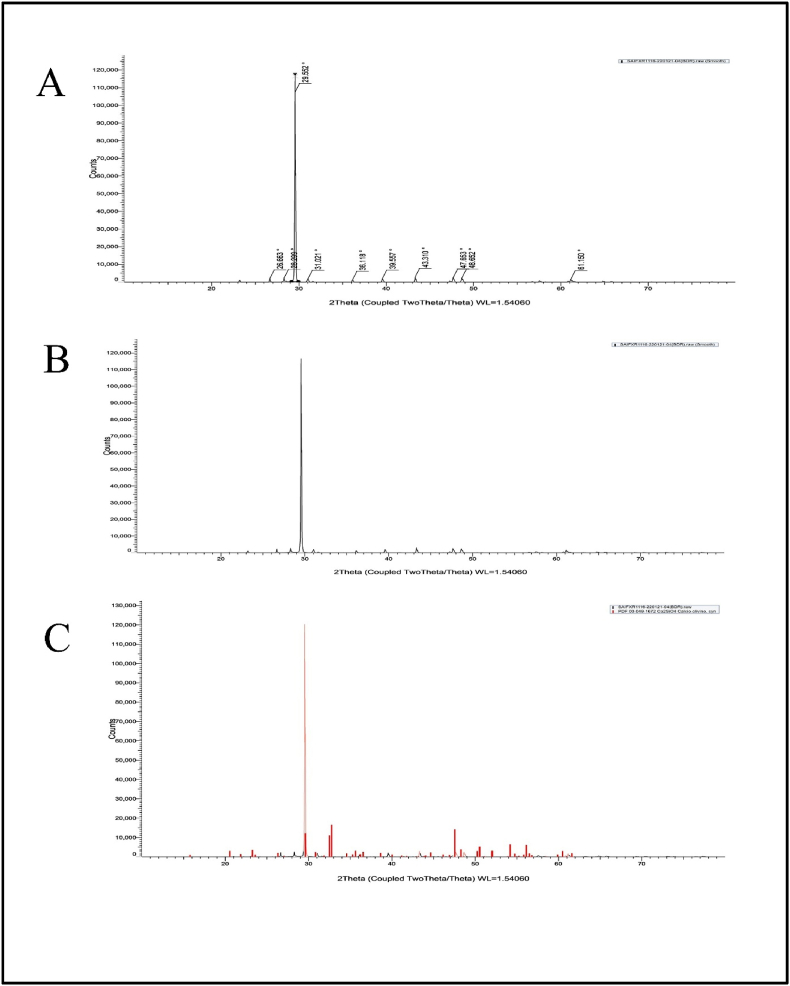
X-ray diffraction pattern of RPL.

**Fig. 7:**
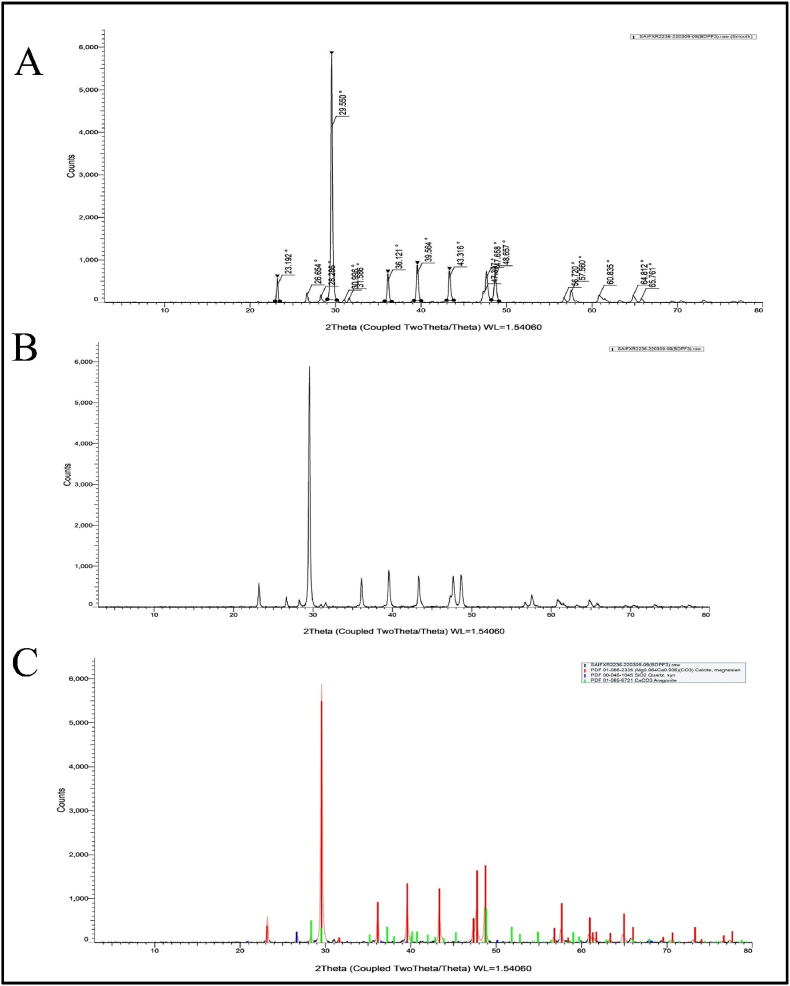
X-ray diffraction pattern of BP.

XRD instrumentation was utilized to figure out the mineralogical composition of the fragments of the RPL samples (Fig. 6) and to do qualitative and quantitative phase analysis of mixtures with more than one phase. The XRD spectral curve shows the presence of CaSiO4 (like calcio-olivine) and quartz in the RPL sample, which was not water soluble in aqueous media. In the XRD curves of BP samples (Fig. 7), the characterization reveals the presence of calcite, magnesium, quartz, aragonite, and calcium carbonate, which were also in corroboration with FTIR studies. Now the question originates: where do the calcite, magnesium, and aragonite come from? When spectral subtractions were used, the spectrum produced the soluble compounds. It was elucidated that after the *Shodhana* (purification by treating with *D. biflorus* L. decoction) and *Bhavana* (wet trituration using rose water) process of raw *Lapis judaicus*, the crystalline nature of these components (calcite, magnesium, and aragonite) retains their identity in a purified micronized form which was loosed in raw form due to disorderedness of crystals. The major reflection intensities of two minerals in both RPL and BP samples were obtained: quartz (26.63) and calcite (29.55), respectively. Furthermore, BP samples include reflections of magnesium (39.54) and aragonite (65.75).

**Fig. 8:**
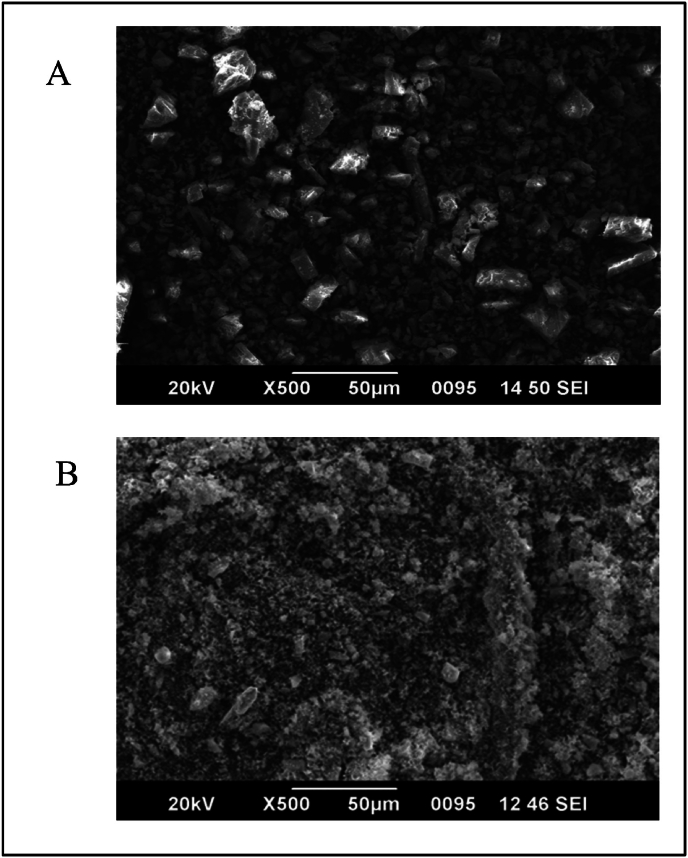
FESEM micrograph image of (A) RPL and (B) BP.

**Fig. 9:**
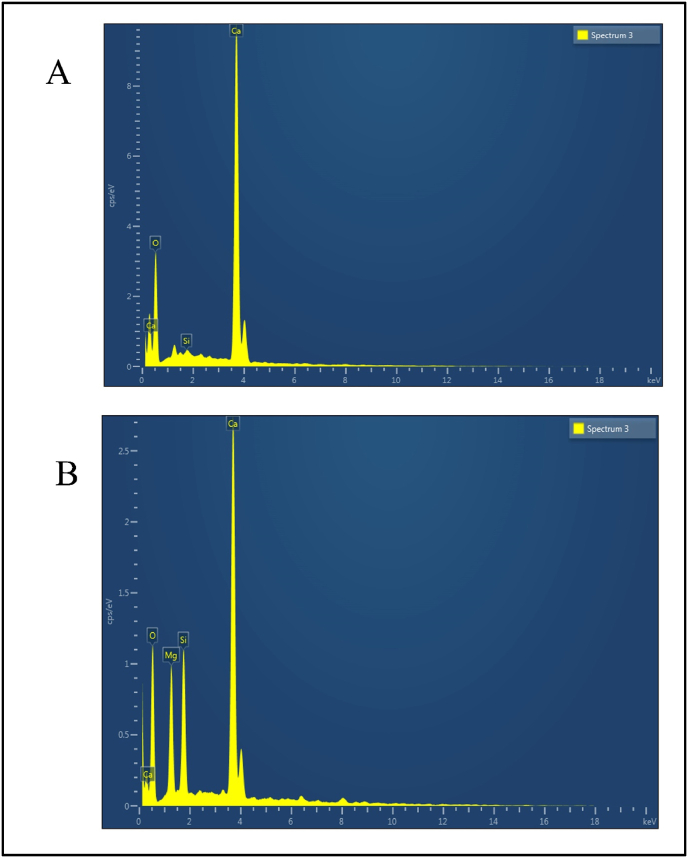
EDAX measurements detect different elements present in (A) the RPL sample and (B) the BP sample.

The size and morphological changes in SEM (scanning electron microscopy) micrographs were distinguishable between the RPL and BP samples in Fig. 8A and B, respectively. The results showed that the particle size of RPL and BP samples were 200–300 μm and 4–10 μm, respectively. In the RPL sample, irregular particle aggregates were seen with anhedral crystals of silicate material. However, in the case of BP, the particles are of micron size with heterogeneously shaped (rhombohedral, hexagonal, irregular) calcite, along with some particles of quartz mineral. Here, we can conclude that the obtained particle size distribution of RPL underwent different transitions during processing. Changes in morphology resulted in the formation of *Lapis judaicus* microparticles. The obtained investigational data may have biomedical applications and help to understand the possible physicochemical and biological behavior of inorganic drugs like *Lapis judaicus*. The EDAX system generates a spectrum that provides both semi-qualitative and semi-quantitative data. The energy, area under the curve, and position of the obtained peak confirm the presence of elements in the sample. The EDAX analysis of the RPL sample revealed the presence of Calcium (Ca), Silica (Si), and oxygen (O) (Fig. 9A). Comparatively, in EDAX analysis of BP samples (Fig. 9B), Calcium (Ca), Magnesium (Mg), Silica (Si) and oxygen (O) were detected. The presence of Mg can be attributed to the treatment of RPL with the *D. biflorus* L. decoction. In a manner analogous to RPL, it has been hypothesized that it might function as a biomedicine as it mostly consists of silica, magnesium, and oxygen. As per observed results, the presence of magnesium is being identified by XRD and EDAX, which inhibits the growth of calcium oxalate crystals. Furthermore, the alkaline nature of *Lapis judaicus* proved to be an additional barrier in renal stone formation. The presence of silicates (SiO_2_) may change the insoluble form of calcium oxalate monohydrate to soluble calcium oxalate dihydrate. The effect of calcium salts in samples on dissolving renal stones is debatable. Previous studies show the promising effects of *Lapis judaicus* in dissolving kidney stones [5,32]. Inorganic drugs have been used since ancient times in various cultures and countries. The use of traditional medicines is limited due to the widespread availability of modern medicinal systems and the lack of sufficient analytical data for standardization, characterization, and physicochemical properties.

## 5 Conclusion

The study prepared and characterized *Badarashma Pishti* and raw powder of *Lapis judaicus*, providing data on their chemical constituents and physicochemical properties. This method could help establish standard procedures for preparing and characterizing other precious gem microparticles used to treat disorders. The maximum dimensions for *Lapis judaicus* microparticles produced through *Bhavana* are significantly smaller compared to those acquired through alternative procedures such as grinding and milling. The pH does not significantly affect the variation in the size of the pores and porous volume for these particles. Ayurvedic textbooks acknowledge the toxicity of heavy metals and suggest specific pharmaceutical methods for their detoxification. Metals and minerals derived from ores often have several impurities. The *Shodhana* method eliminates these contaminants. The *Shodhana* method eliminates undesirable components from the raw material and isolates contaminants. Regarding *Pishti*, the term “*Shodhana*" refers to the process of purifying and preparing the product for the subsequent step, which is *Bhavana*. The prepared *Lapis judaicus* microparticles were analyzed utilizing sophisticated technology, such as FTIR, XRD, SEM-EDAX, and TGA-DSC. Once this study is included in the Ayurvedic Pharmacopoeia of India (API), it can serve as a monograph or regulatory document. It should be noted that EDAX detected trace elements in *Badarashma Pishti* that serve as micronutrients and contribute to therapeutic applications for various diseased conditions that are yet to be explored. According to ancient literature, *Pishti* is believed to possess remarkable medicinal properties if produced using proper standards. Ensuring safety is of utmost importance when developing Herbo-mineral micro or nanoparticles. The ethnomedical practices relating to *Pishti*, the traditional micro or nanoparticles, may be analyzed as a potential prototype to determine whether they may serve as a model.

## Sources of Funding

This work was supported by the CCRAS IMR Project.

## Authors’ contribution

Chandrashekhar Y. Jagtap, Vaibhav Charde, Hemant Rawat, Ganesh Dane, Ashwini Kumar Mishra, Ch. Venkata Narasimhaji: Conceptualization, Formal analysis, Data curation, Validation, Investigation, Writing – original draft, and Writing – review and editing.

Bhagwan S. Sharma, Shruti Khanduri, Ravindra Singh, Narayanam Srikanth, Rabinarayan Acharya: Project administration, Funding acquisition, and Supervision.

## Declaration on use of generative AI in scientific writing

The authors declare that they have not used any generative AI tool or AI assisted technologies while writing this manuscript.

## Conflict of interest

The authors announce that there are no competing interests.
